# Continuous Positive Airway Pressure Reduces Night-Time Blood Pressure and Heart Rate in Patients With Obstructive Sleep Apnea and Resistant Hypertension: The RHOOSAS Randomized Controlled Trial

**DOI:** 10.3389/fneur.2018.00318

**Published:** 2018-05-08

**Authors:** Marie Joyeux-Faure, Jean-Philippe Baguet, Gilles Barone-Rochette, Patrice Faure, Philippe Sosner, Claire Mounier-Vehier, Patrick Lévy, Renaud Tamisier, Jean-Louis Pépin

**Affiliations:** ^1^HP2 Laboratory, INSERM, University Grenoble Alpes, Grenoble, France; ^2^Department of Physiology and Sleep, Grenoble Alpes University Hospital (CHU de Grenoble), Grenoble, France; ^3^Cardiology Department, Grenoble Alpes University Hospital (CHU de Grenoble), Grenoble, France; ^4^Biochemistry, Toxicology and Pharmacology Department, Grenoble Alpes University Hospital (CHU de Grenoble), Grenoble, France; ^5^Cardiology Department, Poitiers University Hospital (CHU de Poitiers) – La Millétrie, Poitiers, France; ^6^Vascular Medicine and Hypertension Department, Lille University Hospital (CHU de Lille), Lille, France

**Keywords:** obstructive sleep apnea, resistant hypertension, leptin, blood pressure, continuous positive airway pressure

## Abstract

**Objective:**

Most patients with resistant hypertension (RH) have obstructive sleep apnea (OSA). We aimed to determine the impact of OSA and continuous positive airway pressure (CPAP) treatment on the leptin profile and blood pressure (BP) in patients with RH.

**Methods:**

After an initial case-control study (RH with and without OSA), we performed a randomized, single blind study in OSA + RH patients receiving either sham CPAP (3 months) followed by active CPAP (6 months) or 6 months of active CPAP. The primary outcome was the comparison of leptin levels between groups of RH patients with or without OSA. Secondary outcomes were the comparison of metabolic parameters, biomarkers of sympathetic activity, and BP indices between the two groups of RH patients with or without OSA. The same outcomes were then evaluated and compared before and after sham and effective CPAP intervention.

**Results:**

Sixty-two patients (60 ± 10 years; 77% men) with RH (24-h daytime systolic BP (SBP)/diastolic BP: 145 ± 13/85 ± 10 mmHg, 3.7 antihypertensive drugs) were included. The 37 RH patients exhibiting OSA (60%) were predominantly men (87 vs 64% for non-OSA patients), with a greater prevalence of metabolic syndrome and higher creatininemia. Their leptin concentrations were significantly lower than in non-OSA patients [9 (6; 15) vs 17 (6; 29) ng/mL] but increased after 6 months of CPAP. Three months of effective CPAP significantly decreased night-time SBP by 6.4 mmHg and heart rate (HR) by 6.0 bpm, compared to sham CPAP.

**Conclusion:**

The association between OSA and RH corresponds to a specific, predominately male phenotype with a higher burden of metabolic syndrome and higher creatininemia but there was no significant difference between OSA and non-OSA patients regarding BP indices, and the number of antihypertensive drugs used. Active CPAP could be efficient at decreasing night-time BP and HR, but there was no difference between CPAP and sham CPAP groups for all metabolic and SNS markers (NCT00746902 RHOOSAS).

## Condensed Abstract

The association between obstructive sleep apnea and resistant hypertension corresponds to a specific, predominately male phenotype with a higher burden of metabolic syndrome and higher creatininemia. Active continuous positive airway pressure decreases night-time blood pressure and heart rate.

## Introduction

Obstructive sleep apnea (OSA) syndrome is characterized by recurrent episodes of upper airway obstruction during sleep, causing intermittent hypoxia (IH) and impaired sleep continuity and quality (1). OSA is recognized as an important and independent risk factor for hypertension (2), coronary heart disease (3), and stroke (4). Resistant hypertension (RH) is defined as blood pressure (BP) that remains above goal in spite of the concurrent use of three antihypertensive agents of different classes (5), and is associated with adverse cardiovascular and renal outcomes and increased mortality (6). OSA is one of the most common causes of RH (7) and OSA patients exhibit a fivefold higher risk of suffering from RH than the general population (8).

In RH the adipokine leptin is associated with lack of BP control primarily mediated by leptin sympathoexitatory effects and renin–angiotensin–aldosterone activation (9, 10) that are also seen in sleep apnea. Adiponectin has been recognized for its antiinflammatory, antiproliferative, and antiatherogenic properties. It has been shown that uncontrolled RH patients have higher leptin and lower adiponectin levels suggesting that abnormal adipokine serum levels contribute to the difficulty in controlling BP in RH (11–13).

Continuous positive airway pressure (CPAP) treatment is the first line therapy for OSA. Recently, two meta-analyses (14, 15) showed that CPAP significantly reduced 24-h BP as well as nocturnal BP in RH patients with OSA (16–19). To date, only one RCT has been sham controlled in a double-blind manner; it confirmed a significant reduction of 24-h systolic BP (SBP) in patients with RH and moderate OSA, after 8 weeks of CPAP treatment (20). The HIPARCO study (17) is by far the largest RCT in the field and has the strength of being multicenter, which allows better generalization of the conclusions. Interestingly, even in the population of CPAP compliers, a significant subgroup did not respond to CPAP treatment (21). This raises the need for a better understanding of the mechanisms underlying RH in this specific OSA population.

To address these issues, we performed a case-control study comparing adipokines and rennin–angiotensin system (RAS) markers in RH patients with or without OSA, followed by a study of the OSA + RH patients who were randomized to receive either sham CPAP or active CPAP.

## Materials and Methods

### Design and Ethics

After a baseline case-control study (RH with OSA vs RH without OSA), we performed a multicenter, randomized, single blind study of the patients with OSA + RH only. Patients were randomized to either sham CPAP (for 3 months) than active CPAP (for 6 months) or active CPAP for 6 months only. The study was conducted in accordance with good clinical practice requirements in Europe, French law, ICH E6 recommendations, and the Helsinki Declaration (1996 and 2000). The protocol was approved by an independent Ethics Committee (Comité de Protection des Personnes, Grenoble, France, IRB0006705) and registered on the ClinicalTrials.gov site (NCT00746902 RHOOSAS). All patients gave their written informed consent.

### Setting and Patients

Consecutive, potentially eligible patients were recruited by the Cardiology departments of 3 University Hospitals (Grenoble, Poitiers and Lille, France) between May 2010 and November 2013. Subjects over 18, with RH and naive of CPAP treatment were eligible. RH was defined as the failure to achieve the target BP (office SBP/diastolic BP (DBP) ≥140/90 or ≥130/80 mmHg for diabetic patients) with three classes of antihypertensive drugs (including diuretics), and 24-h daytime SBP/DBP >135/85 mmHg and/or night-time SBP/DBP >120/70 mmHg.

Patients unable to give written consent or presenting any of the following criteria were not included: known reason for antihypertensive treatment resistance (such as iatrogenic RH, high alcohol consumption, etc.), history of severe renal or cardiac failure or transplantation, Parkinson’s disease, dysautonomia, atrial fibrillation and frequent extrasystoles, severe hypertension (office SBP/DBP ≥180/110 mmHg), daytime alveolar hypoventilation, severe sleepiness defined as the risk of a traffic accident estimated at discretion of investigators, pregnancy, or lactation. Patients with secondary RH were excluded before inclusion.

### Outcomes

The goal of the case-control study was to compare the adipokine profiles in RH patients with or without sleep apnea. This might provide interesting mechanistic insights regarding disease associations and responses to treatment. Other variables compared were BP (office and 24-h), and metabolic, SNS, and RAS biomarkers. In the randomized part of the study, outcomes were evaluated after 3 months of effective CPAP vs sham CPAP, and after 6 months of active CPAP.

### Study Procedure and Variables Measured

At the baseline visit (D0), patients underwent an overnight sleep study as described in Ref. (22) in which apnea–hypopnea index (AHI), mean nocturnal SaO_2_, and time spent with <90% of SaO_2_ were monitored in order to characterize sleep apnea severity, scored according to international guidelines (23). On waking a fasting peripheral blood sample was drawn. The Epworth sleepiness scale was completed and arterial blood gases analysis was performed to exclude obesity hypoventilation syndrome. Patients diagnosed as having OSA (AHI >15/h) were randomized to either CPAP or sham CPAP. Randomization was performed by an independent statistician using a computer-generated randomization. Only patients (but not the study team) were blinded to treatment allocation.

#### BP Measurement Over 24 h

24-h ambulatory BP monitoring (ABPM), which was performed with a Spacelabs 90207 device (Spacelabs Healthcare, Redmond, WA), was measured at baseline for all patients. Measurements were made every 15 min over 24 h. The following ABPM variables were studied: mean HR, SBP, DBP, and MABP [calculated as DBP + 1/3(SBP–DBP)] over the 24 h and over the day (07:00–22:00) and night (22:00–07:00). Patients with a nocturnal reduction in BP compared to average daytime BP of less than 10% were classed as non-dippers and those with more than 10% were classed as dippers. 24-h ambulatory BP measurements with 15 min intervals is the methodology that has been used by the majority of the studies assessing the impact of CPAP on BP in randomized controlled trials (17, 24, 25). We did not make a beat by beat assessment of BP (26) because although it has the advantage of assessing complementary information regarding BP variability this is at the cost of deterioration in sleep quality and duration, and moreover needs to be recorded in the hospital in artificial conditions were different from those of real life.

#### Office BP Measurement

Pressure values were obtained using a mercury sphygmomanometer. The mean of three measurements was calculated on three occasions, in line with European Society of Hypertension–European Society of Cardiology guidelines (27). Office SBP, DBP, and MABP and HR were assessed at baseline for all patients.

#### Metabolic Markers

After peripheral blood sampling, plasma glucose and serum triglyceride concentrations were measured automatically (Dimension Vista 1500, Siemens). Serum insulin was measured using a radio-immunometric sandwich assay (CIS bio international). Serum creatinine was measured using an automated enzymatic system (Dimension Vista 1500, Siemens). The modification of diet in renal disease (MDRD) index was determined according to the literature (28). Ultra-sensitive C-reactive protein (us-CRP) was measured using an automated immunonephelometry technique (Dimension Vista 1500, Siemens). Leptin and adiponectin were measured using a radio-immunometric sandwich assay (MI-HL-81HK kit for leptin and MI-HADP-61HK kit for adiponectin, Millipore). Metabolic syndrome was defined in accordance with the International Diabetes Federation recommendations (29).

#### Sympathetic Nervous System Biomarkers

Methoxylated derivatives of norepinephrine (normetepinephrine) and epinephrine (metepinephrine) were measured in a single blood sample using a high-performance liquid chromatography technique (Clinrep kit, Recipe) coupled with electrochemical detection (colorimetry detector, ESA).

#### Renin–Angiotensin System (RAS) Markers

Aldosterone was measured in a blood sample using a liquid chromatography–tandem mass spectrometry technique, as previously described (30). Active renin was measured using a radio-immunometric sandwich assay (Renin III generation kit, Cisbio).

All markers were measured in duplicate at baseline, after 3 months (3M) of treatment and at the end of the study (M6 or M9).

### Treatments

Active CPAP was provided with an auto-titrating device (Autoset Spirit^®^, ResMed^®^, UK or Remstar Auto^®^, Philips Respironics^®^, Murrysville, PA, USA). Patients receiving sham CPAP had a similar machine delivering a pressure that was too low to maintain the pharynx open. This procedure has been previously validated as an appropriate placebo for CPAP treatment (31). Compliance to active and sham CPAP was recorded by the device. CPAP follow-up was done by experienced homecare providers with nurses making home visits at CPAP initiation and on demand in case of CPAP side effects.

### Sample Size

A sample size calculation was not performed for this study. We included consecutive, eligible, consenting patients with both RH and OSA presenting at the three Cardiology departments between May 2010 and November 2013. Non-OSA RH patients were age matched with patients presenting with OSA. We recognize that we had some difficulties in recruiting eligible patients, resulting in a relatively small sample size despite the long period of inclusion.

### Statistical Analysis

Analysis was performed with SAS software (version 9.4, SAS Institute Inc.). All randomized patients were included in the intention-to-treat (ITT) analysis. The per-protocol population was defined as patients who completed all the visits without any protocol deviation. In the ITT analysis, missing data were replaced by imputation at the median, using the minimum bias method for baseline data and the maximum bias method for data after 3 months of CPAP or sham CPAP treatments. Study design and data are reported here in accordance with the CONSORT criteria (32).

Baseline data were compared by a Student or a Mann–Whitney test for continuous data (depending on the validity of the normality of distribution) and by a Chi^2^ or Fishers exact test for categorical data. A Spearman-rank correlation was used to assess the relationship between leptin concentrations and office DBP.

A *p*-value <0.05 was considered statistically significant. Data are presented as mean ± SD or median [25th; 75th percentiles] while differences (CPAP sham-CPAP active) are presented as mean (95% confidence interval).

For the analysis of changes in values between baseline and 3 months of CPAP or sham CPAP treatment, an analysis of covariance including baseline measurements and the treatment (CPAP/sham CPAP) was performed. The intragroup differences from baseline to 3 months were evaluated with a paired *t*-test or a Wilcoxon test (depending on the validity of the normality of distribution). Logistic regression modeling the probability to be a BP dipper or not was performed.

For the analysis of the evolution in values after 6 months of active CPAP treatment, a mixed model with two factors (fixed factor: group; random factor: time) was performed. A McNemar test was used for dipper probability analysis.

For the treatment effect analysis (with mixed models and Ancova), variables were log transformed when normality was not observed. Because of the similarity of the results between transformed and non-transformed data (and the low values of the residual skewness statistics for the non-transformed data), results were presented as non-transformed data. This is why differences in variables (CPAP-sham CPAP effect) are presented as mean (95% confidence interval). Treatment effect (after 3 and 6 months) was also tested after adjustment to compliance and the interaction between treatment and compliance was analyzed.

## Results

**Figure 1:**
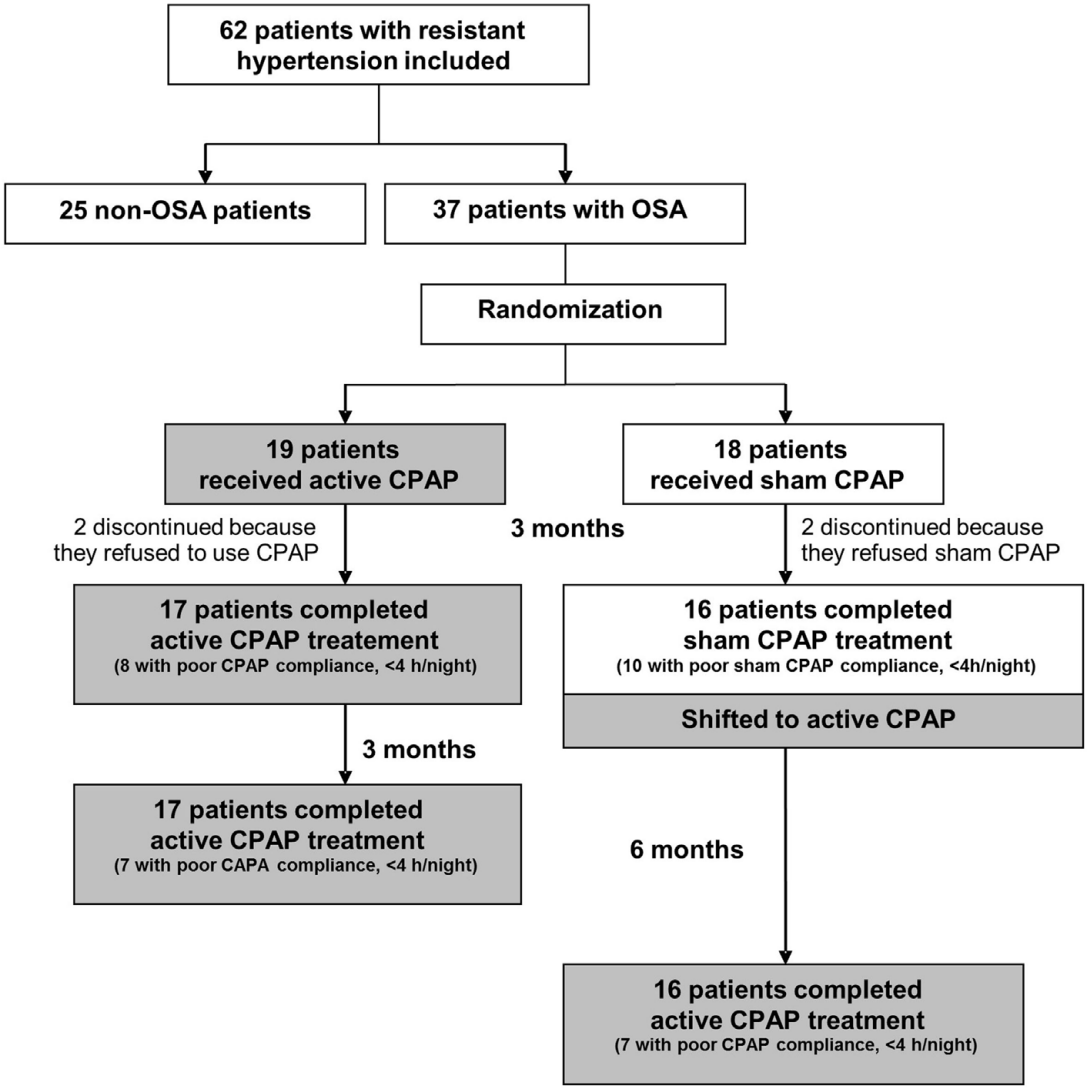
Study flow-chart. Abbreviations: CPAP, continuous positive airway pressure, OSA, obstructive sleep apnea.

The study flow chart is shown in Figure 1.

### Patient Characteristics

Key demographics for the entire study population were: age: 60 ± 10 years, 77% male, mean body mass index (BMI): 29.6 ± 3.9 kg/m^2^, with essential RH (24-h daytime SBP/DBP: 145 ± 13/85 ± 10 mmHg) treated by a mean of 3.7 antihypertensive drugs. Unfortunately, the recruitment rate was lower than expected due to the small number of RH patients assessed at the different cardiology departments and willing to participate. In some centers, the link between sleep centers and cardiology units failed. Also some competitive studies with medications or interventional cardiologic procedures were prioritized.

### Case-Controlled Study Comparing OSA vs Non-OSA Patients With RH

In the 37 apneic patients, 12 had moderate OSA (15/h ≤ AHI < 30/h), 25 severe OSA (AHI ≥ 30/h), and 13 were obese (BMI ≥ 30). Among the 25 non-OSA patients, 11 were obese. Table 1 show that RH patients with OSA were predominantly men and exhibited a significantly higher prevalence of metabolic syndrome.

**Table 1:** Baseline characteristics of patients with resistant hypertension (RH).

	Non obstructive sleep apnea (OSA) patients	OSA patients	*P*-value
*n*	25	37	
Age (years)	58 ± 11	60 ± 9	0.45
Male [% (*n*)]	64.0 (16)	86.5 (32)*	**0.038**
BMI (kg/m^2^)	29.4 [26.5; 31.2]	28.6 [27.1; 31.0]	0.55
Waist circumference (cm)	104 [97; 110]	105 [99; 113]	0.59

**History**			
Smoking [% (*n*)]	52 (13)	57 (21)	0.50
Alcohol [% (*n*)]	32 (8)	30 (11)	0.85
Metabolic syndrome^a^ [% (*n*)]	60 (15)	84 (31)*	**0.036**
Dyslipidemia [% (*n*)]	44 (11)	38 (14)	0.63
MI [% (*n*)]	4 (1)	8 (3)	0.64
Stroke/TIA [% (*n*)]	4 (1)	14 (5)	0.39

**Sleep studies**			
AHI (*n*/h)	8.2 [5.4; 13.2]	37.6 [25.4; 51.8]*	**<0.001**
Mean SaO_2_ (%)	93.5 ± 1.9	92.7 ± 2.0	0.11
SaO_2_ < 90% (%)	0.4 [0.1; 1.3]	5.2 [1.6; 19]*	**<0.001**
Epworth sleepiness scale	9.0 ± 4.4	8.2 ± 4.2	0.53

**Office BP^b^**			
SBP (mmHg)	150 [143; 161]	156 [145; 161]	0.56
DBP (mmHg)	89 ± 14	92 ± 11	0.49
MABP (mmHg)	111 ± 14	112 ± 10	0.63
HR (pbm)	63 [57; 72]	68 [60; 75]	0.12

**24-h BP**			
SBP (mmHg)	140 [135; 148]	139 [133; 150]	0.70
DBP (mmHg)	81.1 ± 9.4	82.8 ± 10	0.50
MABP (mmHg)	101.6 ± 8.7	102.2 ± 9.0	0.79
HR (bpm)	71.0 ± 11.1	69.8 ± 10.3	0.67
Dipper for SBP (%)	32.0 (8)	32.4 (12)	0.97
Dipper for DBP (%)	48.0 (12)	51.4 (19)	0.80

**Antihypertensive drugs**			0.75
3 drugs used [% (*n*)]	44 (11)	51 (19)	
4 drugs used [% (*n*)]	36 (9)	32 (12)	
5 drugs used [% (*n*)]	16 (4)	16 (6)	
Good compliance [% (*n*)]	72 (18)	76 (28)	0.67

**Metabolic markers**			
Total cholesterol (mmol/L)	5.17 [4.14; 5.69]	5.17 [4.40; 5.95]	0.29
LDL cholesterol (mmol/L)	2.84 ± 0.76	3.10 ± 1.03	0.26
HDL cholesterol (mmol/L)	1.29 [1.03; 1.55]	1.03 [1.03; 1.29]	0.06
Triglycerides (mmol/L)	1.24 [1.02; 1.92]	1.69 [1.24; 2.37]*	**0.032**
Fasting glucose (mmol/L)	5.2 [4.9; 6.2]	5.9 [5.4; 7.2]*	**0.018**
Insulinemia (μIU/mL)	10.6 [5.7; 29.0]	12.1 [8.8; 30.8]	0.36
HbA1c	6.0 [5.7; 6.6]	6.0 [5.7; 7.2]	0.72
us-CRP (mg/L)	2.2 [1.0; 4.2]	2.8 [2.0; 5.4]	0.16
Leptin (ng/mL)	17.0 [6.0; 29.0]	9.0 [6.0; 15.0]*	**0.041**
Leptin/weight (ng/mL/kg)	0.22 [0.07; 0.30]	0.10 [0.08; 0.19]*	**0.029**
Adiponectin (ng/mL)	7,820 [4,566; 11,718]	6,393 [4,150; 9,518]	0.26
Creatinine (μmol/L)	78 [67; 86]	90 [82; 104]*	**0.006**
MDRD index (mL/min/1.73 m^2^)	87 ± 21.6	77.6 ± 20.9	0.09

**SNS markers**			
Normetepinephrine (nmol/L)	10.1 [6.2; 12.3]	10.0 [8.8; 16.6]	0.27
Metepinephrine (nmol/L)	4.0 [2.8; 5.1]	4.6 [3.5; 5.5]	0.42

**SRA markers**			
Aldosterone (ng/L)	134 [77; 258]	100 [54; 165]	0.14
Renin (ng/L)	11.8 [5.4; 16.0]	15.3 [6.9; 41.9]	0.17

*Data are mean ± SD, median [25th; 75th percentiles] or percentage*.

*^a^Metabolic syndrome was defined according to the International Diabetes Federation recommendations (29)*.

*^b^Ambulatory BP monitoring was mandatory to define RH. Office BP values are provided only for readers information*.

*p < 0.05 by Student or Mann–Whitney test. For qualitative data, a Chi-square test or a Fisher test was performed.

*HbA1c, glycosylated hemoglobin; AHI, apnea–hypopnea index; BMI, body mass index; BP, blood pressure; DBP, diastolic blood pressure; HDL, high-density lipoprotein; HR, heart rate; LDL, low-density lipoprotein; MABP, mean arterial BP; MDRD, modification of diet in renal disease; MI, myocardial infarction; RAS, renin–angiotensin system; SaO_2_, oxygen saturation; SaO_2_ < 90%, percentage of recording time spent at a SaO_2_ < 90%; SBP, systolic blood pressure; SNS, sympathetic nervous system; TIA, transient ischemic attack; us-CRP, high-sensitivity C-reactive protein*.

*Bold font indicates the significativity of the results*.

There was no significant difference between OSA and non-OSA patients regarding other baseline demographic data, medical history, baseline office BP, 24-h BP, and the number of antihypertensive drugs used (Table 1). Antihypertensive medication in the different patient groups is reported in Table 2.

**Table 2:** Antihypertensive medications in the different patient groups.

Different antihypertensive classes	Non obstructive sleep apnea (OSA) patients *n* = 25	OSA patients	
		Continuous positive airway pressure (CPAP) *n* = 19	Sham CPAP *n* = 18
Centrally acting antihypertensive drugs	4 (16)	1 (5)	2 (11)
α1-Blockers	7 (28)	5 (26)	2 (11)
β-Blockers	13 (52)	8 (42)	11 (61)
Diuretics	25 (100)	18 (95)	18 (100)
Angiotensin-converting enzyme inhibitors	9 (36)	6 (32)	7 (39)
Calcium channel blockers	23 (92)	15 (79)	14 (78)
Renin blockers	4 (16)	2 (11)	1 (6)
Angiotensin II receptor blockers	10 (40)	12 (63)	10 (56)
Peripheral vasodilators	0 (0)	1 (5)	0 (0)

*Data are number of patients (percentage)*.

In patients with RH, we observed significantly lower leptin concentrations in the presence of OSA, independent of the patients’ weight (Table 1) and OSA severity (data not shown). An inverse correlation between leptin concentrations and office DBP (−0.349, *p* = 0.006) and MABP (−0.277, *p* = 0.029) was found for the whole group.

Fasting glucose, triglycerides, and creatinine levels were significantly higher in RH patients with OSA (Table 1). All other metabolic, SNS and RAS markers were comparable between OSA and non-OSA patients.

### Comparison Between 3 Months of Active CPAP and Sham CPAP Treatment in RH Patients With OSA

#### After Randomization and Before CPAP Treatment

After randomization and before CPAP treatment, there was no significant difference between the CPAP (*n* = 19) and the sham CPAP (*n* = 18) groups regarding all demographic data, medical history, respiratory and biological markers, BP (Table 3), and the number of antihypertensive drugs used. In both groups, two patients discontinued because they refused to use the treatment (but not because they felt the treatment was not effective). There are many previous studies using a sham CPAP design that did report a placebo effect in the sham CPAP arm (33). After 3 months, mean treatment compliance was comparable between CPAP [3.90 (0.60; 5.82) h/night] and sham CPAP [1.86 (0.60; 6.75) h/night] groups, as well as the number of compliant patients/group (use >4 h/night, Figure 1).

**Table 3:** BP at baseline (D0) and after 3 months (M3) of effective continuous positive airway pressure (CPAP) or sham CPAP treatment in patients with resistant hypertension (RH) and obstructive sleep apnea (OSA).

	CPAP (*n* = 19)			Sham CPAP (*n* = 18)			Difference in data change (CPAP-sham CPAP effect)^a^	*P*-value

	D0	M3	*P*-value	D0	M3	*P*-value
**Office BP**								
SBP (mmHg)	149 [144; 159]	143 [133; 155]	0.11	158 [151; 164]	152 [139; 169]	0.38	−1.18 (−11.37; 9.01)	0.82
DBP (mmHg)	90 [78; 98]	89 [83; 93]	0.19	94 [83; 102]	91 [78; 104]	0.93	−3.08 (−10.41; 4.26)	0.40
MABP (mmHg)	109 [102; 118]	109 [100; 110]	0.08	116 [108; 120]	108 [102; 124]	0.75	−2.15 (−9.45; 5.14)	0.55
HR (bpm)	66 [60; 71]	63 [60; 69]*	**0.046**	73 [61; 87]	66 [60; 73]	0.12^b^	0.52 (−4.51; 5.55)	0.83

**24-h BP**								
SBP (mmHg)	142 [137; 152]	138 [135; 141]*	**0.034**	137 [130; 148]	137 [131; 143]	0.59	−1.88 (−7.91; 4.16)	0.53
DBP (mmHg)	84 [78; 90]	80 [74; 86]*	**0.011**	81 [75; 91]	78 [71; 88]	0.20	−1.69 (−6.16; 2.78)	0.45
MABP (mmHg)	102 [97; 111]	97 [94; 105]*	**0.019**	98 [94; 109]	98 [91; 103]	0.30	−1.55 (−6.45; 3.35)	0.52
HR (bpm)	73 [64; 76]	70 [62; 72]	0.12	66 [61; 73]	69 [64; 77]	0.24^b^	−3.38 (−7.61; 0.84)	0.11

**Daytime BP**								
SBP (mmHg)	145 [139; 155]	141 [138; 148]	0.46	139 [132; 151]	139 [134; 147]	0.56	1.29 (−5.91; 8.48)	0.72
DBP (mmHg)	87 [82; 94]	83 [78; 92]	0.30	84 [77; 94]	83 [73; 90]	0.44	−0.15 (−5.71; 5.41)	0.96
MABP (mmHg)	107 [99; 114]	102 [98; 111]	0.37	103 [95; 111]	102 [97; 106]	0.47	0.33 (−5.70; 6.36)	0.91
HR (bpm)	74 [65; 82]	72 [65; 78]	0.41	69 [63; 82]	71 [66; 81]	0.46	−1.69 (−6.55; 3.17)	0.48

**Night-time BP**								
SBP (mmHg)	132 [128; 145]	127 [122; 134]*	**0.002**	131 [124; 139]	127 [125; 137]	0.82	−6.37 (−12.18; −0.55)	**0.033**
DBP (mmHg)	75 [72; 83]	70 [66; 76]*	**0.002**	77.0 [68; 85]	73 [65; 80]*	**0.022**^b^	−2.50 (−6.91; 1.90)	0.26
MABP (mmHg)	95 [91; 102]	92 [86; 94]*	**0.002**	96 [85; 103]	91 [85; 98]	0.05^b^	−3.14 (−7.83; 1.56)	0.18
HR (bpm)	65 [59; 70]	63 [57; 66]*	**0.016^b^**	59.5 [57; 66]	63 [60; 72]	0.10^b^	−5.98 (−10.45; −1.52)	**0.010**

**Dipper profile**								
for SBP [% (*n*)]	36.8 (7)	47.4 (9)	0.48	27.8 (5)	33.3 (6)	0.56	1.67 (0.40; 6.94)	0.48
for DBP [% (*n*)]	52.6 (10)	47.4 (9)	0.74	50.0 (9)	33.3 (6)	0.39	1.88 (0.45; 7.89)	0.39

*Data are mean ± SD or median [25th; 75th percentiles] or percentage*.

*^a^Mean (95% confidence interval), adjusted on baseline values, negative values = higher CPAP effect*.

*^b^Non-parametric tests used due to the non-normality of the distribution*.

*p < 0.05 vs data at D0.

*BP, blood pressure; HR, heart rate; SBP, systolic blood pressure; DBP, diastolic blood pressure; MABP, mean arterial pressure*.

*Bold font indicates the significativity of the results*.

#### BP Analysis

In ITT analysis, 3 months of CPAP treatment significantly lowered night-time SBP and heart rate (HR) compared to sham CPAP (Table 3). Moreover, CPAP significantly decreased night-time DBP [by 8.10 (−13.91; −2.29) mmHg, *p* = 0.009] and MABP [−9.09 (−16.75; −1.43) mmHg, *p* = 0.023], compared to sham CPAP, but only in patients with baseline values ≥ the median. Finally, office BP, 24-h BP and dipper profile were not modified by 3 months of CPAP treatment, compared to sham CPAP. The results were the same after adjustment for CPAP compliance, which was comparable between arms. Results were similar in a per-protocol analysis.

#### Biological Markers

After 3 months of treatment, there was no difference between CPAP and sham CPAP groups for all metabolic and SNS markers, with the exception of aldosterone serum levels that were significantly increased after 3 months of sham CPAP compared with active CPAP, whereas renin was not modified (Table 4). These results were the same in ITT analysis adjusted for CPAP compliance and per-protocol analysis (before and after adjustment for CPAP compliance and gender).

**Table 4:** Biological markers at baseline (D0) and after 3 months (M3) of effective continuous positive airway pressure (CPAP) or sham CPAP treatment for patients with resistant hypertension (RH) and obstructive sleep apnea.

	CPAP (*n* = 19)			Sham CPAP (*n* = 18)			Difference in data change (CPAP-sham CPAP effect)^a^	*P*-value
	D0	M3	*P*-value	D0	M3	*P*-value		
**Metabolic markers**								
Total cholesterol (mmol/L)	5.17 [4.40; 5.95]	5.43 [4.40; 5.95]	0.35	5.95 [4.11; 6.05]	5.35 [4,47; 5.82]	0.82	0.00 (−0.20; 0.19)	0.97
LDL cholesterol (mmol/L)	3.28 [2.59; 4.09]	3.15 [2.66; 3.88]	0.89^b^	3.36 [2.59; 3.62]	3.10 [2.59; 3.62]	0.34	0.09 (−0.07; 0.24)	0.27
HDL cholesterol (mmol/L)	1.03 [1.03; 1.29]	1.29 [1.03; 1.55]	0.28	1.19 [0.88; 1.34]	1.19 [1.01; 1.37]	0.17^b^	0.02 (−0.07; 0.11)	0.71
Triglycerides (mmol/L)	1.69 [1.13; 2.37]	1.69 [1.02; 2.15]	0.20	1.99 [1.29; 2.57]	1.79 [1.10; 2.52]	0.80^b^	−0.30 (−0.76; 0.16)	0.20
Fasting glucose (mmol/L)	5.9 [5.1; 6.9]	5.8 [5.1; 6.6]	0.37	6.0 [5.9; 7.3]	6.2 [5.2; 7.3]	0.43^b^	0.17 (−1.98; 2.32)	0.87
Insulinemia (μIU/mL)	18.8 [8.5; 31.0]	9.6 [5.7; 22.0]	0.12	11.4 [8.8; 30.1]	9.5 [7.7; 14.9]*	**0.018**	1.77 (−4.40; 7.95)	0.56
HbA1c	5.9 [5.7; 7.2]	5.9 [5.7; 6.6]	0.71	6.0 [5.8; 7.6]	5.9 [5.8; 6.7]	0.59	0.22 (−0.31; 0.76)	0.40
Creatininemia (μmol/L)	95.0 [86.0; 103.0]	92.0 [84.0; 107.0]	0.22^b^	87 [76; 109]	91 [77; 98]	0.84^b^	4.63 (−3.26; 12.52)	0.24
MDRD index (mL/min/1.73 m^2^)	73 [69; 86]	76 [64; 86]	0.59^b^	77 [62; 93]	76 [59; 90]	0.81	−0.57 (−7.01; 5.87)	0.86
Leptin (ng/mL)	7.0 [4.0; 18.0]	10.0 [6.0; 18.0]	0.20	10.0 [8.0; 15.0]	12.0 [9.0; 14.0]	0.40^b^	−0.03 (−5.04; 4.99)	0.99
Leptin/weight (ng/mL/kg)	0.09 [0.05; 0.17]	0.13 [0.07; 0.16]	0.31	0.10 [0.08; 0.19]	0.13 [0.10; 0.19]	0.38^b^	−0.01 (−0.06; 0.04)	0.60
Adiponectin (ng/mL)	7,004 [5,162; 10,181]	6,560 [5,747; 9,845]	0.80	5,134 [3,846; 9,276]	7,104 [4,428; 8,409]*	**0.030**	−2761 (−7415; 1,892)	0.24

**SNS markers**								
Normetepinephrine (nmol/L)	10.0 [8.3; 16.6]	9.3 [6.5; 10.5]	0.05	10.0 [8.8; 19.1]	9.2 [7.4; 14.4]	0.12^b^	−0.31 (−3.87; 3.25)	0.86
Metepinephrine (nmol/L)	4.7 [3.2; 5.5]	3.9 [3.0; 6.8]	0.69^b^	4.4 [3.8; 5.1]	4 [2.4; 4.4]	0.15^b^	0.69 (−0.63; 2.02)	0.29

**SRA markers**								
Aldosterone (ng/L)	78 [41; 220]	93 [68; 141]	0.54^b^	103 [72; 154]	140 [101; 215]*	**0.038**^b^	−58.3 (−101.7; −14.9)	**0.010**
Renin (ng/L)	11.1 [7.2; 37.9]	8.2 [5.8; 35.9]	0.34	16.3 [6.6; 58.9]	9.7 [4.9; 39.7]	0.06	3.66 (−14.50; 21.82)	0.68

*Data are mean ± SD or median [25th; 75th percentiles]*.

*^a^Mean (95% confidence interval), adjusted on baseline values negative values = higher sham CPAP effect*.

*^b^Parametric test used due to the normality of the distribution*.

*p < 0.05 vs data at D0.

*HbA1c, glycosylated hemoglobin; HDL, high-density lipoprotein; LDL, low-density lipoprotein; MDRD, Modification of Diet in Renal Disease; RAS, renin–angiotensin system; SNS, sympathetic nervous system*.

*Bold font indicates the significativity of the results*.

### Effect of 6 Months of Active CPAP Treatment in RH Patients With OSA

In ITT analysis, night-time BP significantly decreased after 6 months of active CPAP treatment [SBP: −4.7 (8.4; −0.9) mmHg (*p* = 0.016) and DBP: −2.3 [−4.5; −0.2] mmHg (*p* = 0.036)]. Moreover, dipper profile was improved by active CPAP [56.8 vs 29.7% (*p* = 0.004) for SBP, and 64.9 vs 48.7% (*p* = 0.058) for DBP]. After 6 months of active CPAP treatment, blood leptin concentrations increased significantly, independently of the patient’s weight [Leptin/weight: 0.04 (0.00; 0.07) ng/mL/kg (*p* = 0.025)].

The amount of missing data was between 5 and 16% for data before and after CPAP treatment.

## Discussion

The originality of our study was to address both the hormonal and metabolic characteristics of the combination of OSA and RH and the effect of CPAP in a randomized sham CPAP controlled trial. OSA patients with RH exhibited distinct characteristics compared to non-OSA RH. They were more often men with a higher prevalence of metabolic syndrome and significantly lower leptin concentrations than in non-OSA patients. Three months of active CPAP significantly decreased night-time SBP by 6.4 mmHg (*p* = 0.033) and HR by 6.0 bpm (*p* = 0.010) compared to sham CPAP. The presence of OSA in 60% of the included RH patients is in line with previous studies (7, 8, 34). The negative impact of OSA on renal function has now been demonstrated both in animal models and in epidemiological studies (35), and accordingly our study found higher levels of creatinine in the group with OSA and RH. Thus, systematic screening for sleep apnea is one of the more prevalent and treatable secondary causes of RH are now recommended by the European society of Hypertension (36).

We observed that compared to non-OSA RH patients, metabolic syndrome was more prevalent in those with OSA, with significantly higher triglyceridemia and fasting glucose levels. OSA is known to be associated with a ninefold higher risk of metabolic syndrome after adjustment for confounders (37, 38). As metabolic syndrome and renal function are major predictors of late cardiovascular events this suggests that the association of OSA and RH forms a specific high-risk subgroup.

High leptin levels are associated with impaired BP control and poor long-term prognosis (39, 40). There remains a controversy as to the relationship between circulating leptin levels and OSA, since several studies have demonstrated higher leptin levels in subjects with OSA compared with BMI-matched control subjects, suggesting a relative leptin-resistant state in OSA (41–44), whereas in other studies the relationship was suppressed after adjustment for obesity (45, 46). At baseline, we found counter-intuitive lower leptin concentrations (independent of patient weight) in OSA patients with RH, that were inversely correlated with office DBP and MABP. OSA patients were predominantly males (87% compared to 64% for non-OSA) and leptin concentrations are known to be lower in males (46), thus the lower leptin concentrations observed in the OSA group could be partly attributed to different sex ratios in the two populations. In healthy humans, Spiegel et al. showed that sleep deprivation lowered leptin levels and blunted their normal diurnal variation (47–50). Sleep deprivation and short sleep duration are common in RH (51) and could contribute toward lower leptin levels. Furthermore, Patel et al. have suggested that OSA may selectively suppress morning secretion of leptin with a relative elevation in leptin level in the evening. Relatively low morning levels may contribute to increased morning appetite and weight gain in individuals with OSA (46). We showed that before treatment, leptin levels were significantly lower in patients with both OSA + RH compared to RH alone. In accordance with Mark et al. (52), in this situation the level of sympathetic activation might be higher inhibiting leptin secretion by the adipocytes. This could be partly reversed by CPAP treatment.

In accordance with a previous study (7), we observed no difference regarding office and 24-h BP, or in dipping pattern, between the OSA and non-OSA patients with RH suggesting that the presence of OSA has a limited impact on BP control in RH patients. However, we found that in these patients 3 months of active CPAP treatment lowered night-time BP (in particular SBP) compared to sham CPAP, without modifying daytime and 24-h BP. This fall in night-time BP was confirmed after 6 months of active CPAP treatment that also improved dipper profile. Our results are in line with a meta-analysis (15) that included four RCTs examining the effect of CPAP vs no CPAP (16–19) and one RCT using sham CPAP (20) and showed that overall CPAP treatment reduces night-time BP, but not daytime BP, in patients with OSA and RH. Indeed, nocturnal BP levels are a better predictor of cardiovascular risk than daytime BP levels (53). Therefore, nocturnal BP should be controlled to reduce the risk of cardiovascular disease. We also showed that 3 months of CPAP treatment lowered night-time HR, compared to sham CPAP, in OSA patients with RH. As ambulatory HR is a predictor of cardiovascular mortality in hypertensive patients and that lowering HR is essential for prevention (54, 55), CPAP treatment would provide additional benefits for RH patients.

### Study Limitations

The primary outcome was the comparison of leptin levels in the case-control study. We acknowledge that the study essentially provides descriptive data and mechanistic insights that will facilitate the sample size calculation and design of further studies. We did not make an *a priori* sample size calculation. We recognize that we had some difficulties in recruiting eligible patients, resulting in a relatively small sample size. Second, we did not have access to an objective measurement of adherence to antihypertensive medications, which is a major cause of suboptimal BP control. This poor compliance behavior commonly seen in RH patients is certainly part of the explanation for poor CPAP adherence. Indeed, we acknowledge that CPAP adherence was low both in the effective and in the sham CPAP arms. This is a frequent finding in studies in the OSA field (56). This reflects the real life situation and is accounted for by the intention to treat and per-protocol analyses. Our data give an indication of the range of BP reduction that can be expected in an unselected population with OSA plus RH. Lastly, as some patients were characterized by respiratory polygraphy, we were not able to determine the effect of active CPAP on the arousal index. We speculate that RH itself is associated with persistent sympathetic over-activity that conceals the CPAP effect. Another limitation is that sympathetic nervous system biomarkers have been evaluated in a single blood sample and not in a 24-h urine sample as recommended. Finally, the 6-month effective CPAP study was observational and we acknowledge a potential heterogeneity as some patients were truly CPAP naïve, whereas other patients had been treated with sham CPAP (3 months).

In conclusion, the association between OSA and RH corresponds to a specific phenotype with male predominance, a higher burden of metabolic syndrome and higher levels of creatinine. Active CPAP could be efficient at decreasing night-time BP and HR.

## Author Note

All authors take responsibility for all aspects of the reliability and freedom from bias of the data presented and their discussed interpretation.

## Ethics Statement

The study was conducted in accordance with good clinical practice requirements in Europe, French law, ICH E6 recommendations, and the Helsinki Declaration (1996 and 2000). The protocol was approved by an independent Ethics Committee (Comité de Protection des Personnes, Grenoble, France, IRB0006705) and registered on the ClinicalTrials.gov site (NCT00746902 RHOOSAS). All patients gave their written informed consent.

## Author Contributions

MJ-F and J-LP: designed the study, collected data, contributed to discussion, wrote the manuscript, and reviewed/edited the manuscript. J-PB, GB-R, PF, PS, CM-V, PL, and RT: included patients, collected data, and or performed analyses, contributed to the discussion, and reviewed/edited the manuscript.

## Conflict of Interest Statement

The authors declare that the research was conducted in the absence of any commercial or financial relationships that could be construed as a potential conflict of interest.
